# A Drug Utilization Study of Anti-glaucoma Drugs in a Tertiary Care Hospital

**DOI:** 10.7759/cureus.46765

**Published:** 2023-10-09

**Authors:** Anusha Meesala, Ratikanta Tripathy, Harish Chandra Chaudhury, Jyoti Prakash Sahoo

**Affiliations:** 1 Pharmacology, Andhra Medical College, Visakhapatnam, IND; 2 Pharmacology, Kalinga Institute of Medical Sciences, Bhubaneswar, IND

**Keywords:** polytherapy, fixed-dose combination, carbonic anhydrase inhibitors, prostaglandin, beta-blockers, open angle glaucoma

## Abstract

Background and objectives: Today, branded medications and polytherapy are frequently prescribed for glaucoma, even without giving the patient the proper instructions. Hence, the safety, effectiveness, cost, and patient compliance of glaucoma medication must be weighed, and the anti-glaucoma medicine usage must be studied. Analysis of glaucoma patients' prescription usage was the objective of this study.

Material and methods: Between January 2021 and February 2022, this prospective and observational study was carried out at Andhra Medical College in Vishakhapatnam. One hundred prescriptions of those with primary open-angle and angle closure glaucoma were assessed. Age and gender-based subgroup analyses were conducted. R software (version 4.2.1) (The R Foundation for Statistical Computing, Vienna, Austria) was leveraged for data analysis.

Results: Out of 146 examined prescriptions, 100 (69%) were deemed suitable for analysis. Participants' mean age was 54.2 ± 10.8 years. Sixty-two were over 50 years old, and 36 were men. The mean intraocular pressure was 25.4 ± 1.7 mm of Hg. Per prescription, there were about 1.75 anti-glaucoma drugs. Fixed-dose combinations (FDC) were found in 43 prescriptions. Generic medications and patient instructions prevailed in most prescriptions (78%) and (84%). Timolol was used in each FDC with brimonidine, dorzolamide, or bimatoprost.

Conclusion: The most often prescribed anti-glaucoma drug, timolol, was also identified as an essential component of the FDC. Doctors must prescribe generic medications with detailed directions for the patients.

## Introduction

The prescribing, marketing, distribution, and use of medications in a society, with an eye on the resultant medical, social, and economic repercussions, constitute drug utilization studies, according to the World Health Organization (WHO) [1]. The usefulness of hospital formularies can be weighed through drug utilization research studies in the outpatient and inpatient contexts. The usage of pharmaceuticals varies perpetually across countries, between healthcare facilities in the same country, and occasionally within a single institution at various junctures in time, presumably as a consequence of shifting disease patterns with time [2]. In developing countries like India, such studies are necessary to ensure that the limited resources are utilized as efficiently as possible.

Though various drug utilization studies have been conducted regarding anti-glaucoma drugs in India [3-7], only a few have done detailed analyses [8,9]. Age and gender-based variations regarding the epidemiology of glaucoma have prevailed for decades. Elderly females are more prone to have various types of glaucoma irrespective of their comorbidities [10,11]. To date, no studies emphasizing drug utilization of fixed-dose combinations (FDC) and polytherapy in glaucoma patients have been carried out in the Indian subcontinent. Considering these facts, we planned this study to determine the drug utilization pattern by analyzing prescriptions of glaucoma patients.

## Materials and methods

This observational study went ahead from January 2021 to February 2022 at Andhra Medical College, Vishakhapatnam. Before study initiation, we got approval (serial no. 16/IEC AMC/JAN/2021) from the Institutional Ethics Committee of Andhra Medical College, Vishakhapatnam, Andhra Pradesh. Prior to the individuals' enrollment, their consent was sought. Study participants were adults (> 18 years of age) with glaucoma and intraocular pressure (IOP) of more than 23 mm of Hg. The persons with acute angle closure glaucoma, any cardiovascular, metabolic, or endocrine disorders, chronic liver disease, renal impairment with estimated glomerular filtration rate (eGFR) ≤ 30 ml/min/m^2^, tuberculosis, hepatitis, HIV, and pregnant or nursing women were excluded from the study. The primary objective was determining the number and proportions of patients prescribed with various anti-glaucoma medications. The secondary objectives were determining the number and proportions of patients prescribed with monotherapy, polytherapy, and FDC.

Convenience sampling was adopted for this study. After noting down the required parameters, prescriptions were returned to the patients. All the prescriptions were analyzed for the objectives mentioned above. The categorical and continuous variables were expressed in number (percentage) and mean ± standard deviation. The chi-square test was applied. The study population was categorized per gender (i.e., female and male) and age group (i.e., ≤ 50 and > 50 years) to determine the gender and age-based variations in the prescriptions. For the computation of the data, we used the R software (version 4.2.1) (The R Foundation for Statistical Computing, Vienna, Austria) [12].

## Results

In this study, we examined 146 prescriptions of patients who had either primary open angle or angle closure glaucoma. Twenty-three patients had acute angle closure glaucoma, 10 were taking multiple antihypertensives, eight had uncontrolled diabetes, and five refused to provide their consent. Finally, 100 (68.5%) prescriptions (36 men and 62 elderly patients) were examined (Table 1). Participants' mean age was 54.2 ± 10.8 years. The mean IOP was 25.4 ± 1.7 mm Hg. Per prescription, there were approximately 1.75 anti-glaucoma drugs (95% CI: 1.04-2.47). Generic medications and patient instructions were present in most of these prescriptions (i.e., 78% and 84%, respectively).

**Table 1:** Demographic parameters and drug-use indicators of study participants (n = 100) The categorical and continuous data were expressed as n (%) and mean ± standard deviation. BMI: body mass index, CI: confidence interval, FDC: fixed-dose combination

Parameters	Values
Age (years)	
Mean age	54.2 ± 10.8
Median (range)	53 (35-85)
Male, n (%)	36 (36.0%)
> 50 years, n (%)	62 (62.0%)
Weight (kg)	49.7 ± 11.4
BMI (kg/m^2^)	23.3 ± 3.1
Primary open-angle glaucoma	93 (93.0%)
Intraocular pressure (mm of Hg)	25.4 ± 1.7
Average drug per prescription (95% CI)	1.75 (1.04-2.47)
Prescriptions with monotherapy	39 (39.0%)
Prescriptions with FDC	43 (43.0%)
Prescriptions with generic drugs only	78 (78.0%)
Prescriptions mentioning dosing frequency and duration	84 (84.0%)

**Figure 1:**
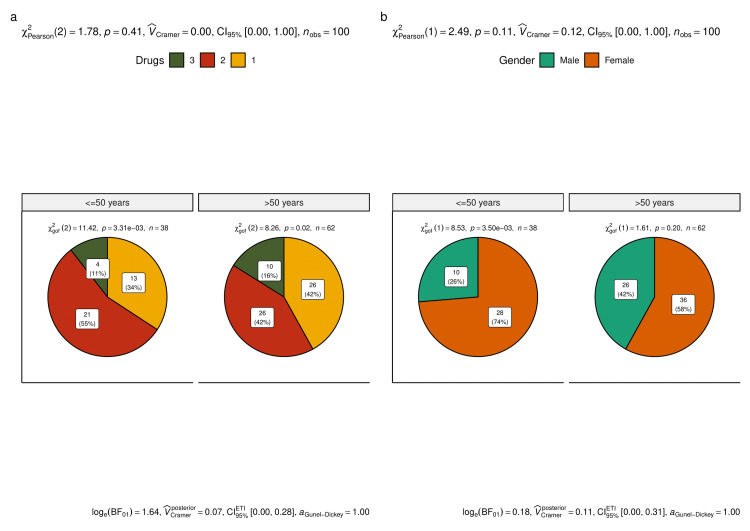
Drug utilization and demographics of the study participants (based on age group) The pie diagrams illustrate the demographics of the study participants (based on age group) pertaining to the anti-glaucoma drugs. Figure 1a shows the number (proportion) of younger (≤ 50 years) and elderly (> 50 years) individuals prescribed with a number of anti-glaucoma drugs. Figure 1b shows the number (proportion) of male and female participants in the two age groups.

Figure 1 shows the participant demographics and drug utilization pattern based on the age group of the participants (i.e., younger (≤ 50 years of age) and elderly (> 50 years of age)). Figure 1a shows the number (proportion) of younger and elderly individuals prescribed with a number of anti-glaucoma drugs. Intergroup comparison yielded non-significant differences (p = 0.41) whereas intragroup comparisons revealed statistically significant differences. Figure 1b shows the number (proportion) of male and female participants in the two age groups. Intergroup comparison gave a non-significant difference (p = 0.11).

**Figure 2:**
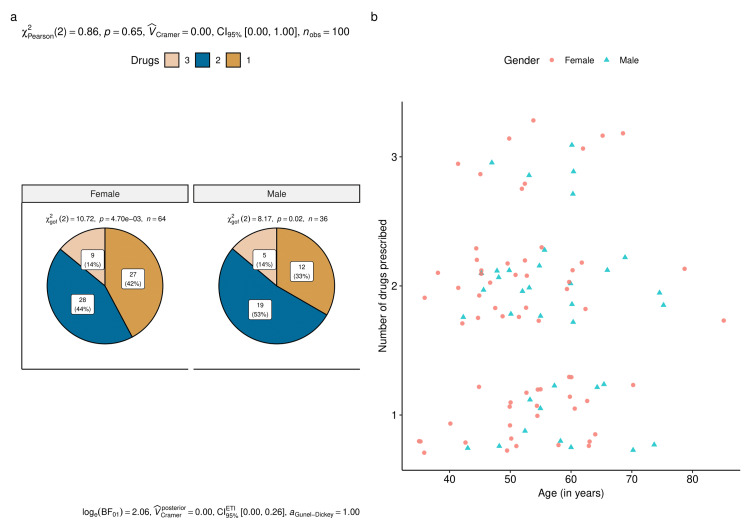
Drug utilization among the study participants (based on gender) The figures illustrate the number of prescribed anti-glaucoma drugs to the female and male participants. The pie diagram in Figure 2a shows the number (proportion) of females and males prescribed with a number of anti-glaucoma drugs. The jitter plots in Figure 2b show the number of anti-glaucoma drugs prescribed to all the study participants.

Figure 2 shows the prescription pattern based on the gender of the participants. Figure 2a shows the number (proportion) of female and male participants prescribed with a number of anti-glaucoma drugs. Intergroup comparison showed a non-significant difference (p = 0.65) whereas intragroup comparisons were statistically significant. Figure 2b displays each person's gender and age in conjunction with the number of prescribed medications.

**Figure 3:**
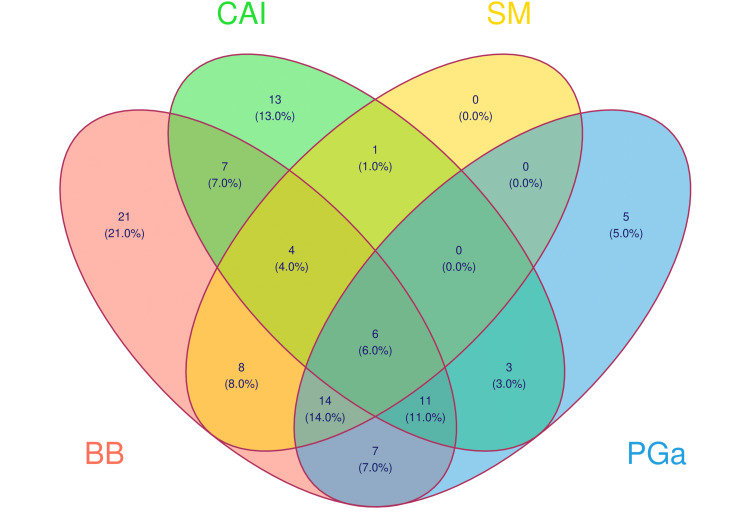
Venn diagram of prescription pattern among the study participants The Venn diagram shows the number (proportion) of the study participants prescribed with various anti-glaucoma drugs. BB: beta-blockers, CAI: carbonic anhydrase inhibitors, SM: alpha-1 sympathomimetics, PGa: prostaglandin analogs

**Figure 4:**
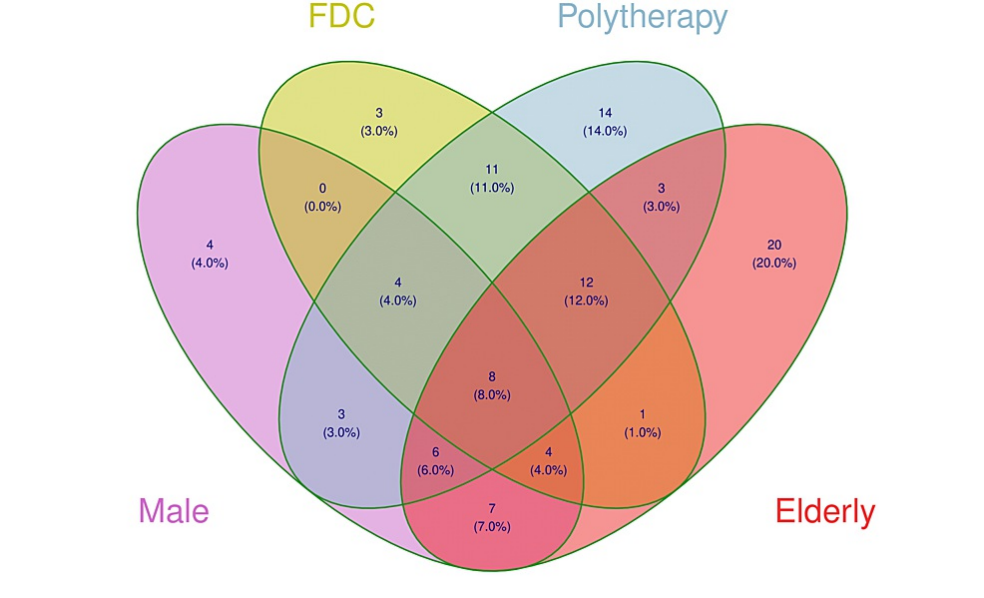
Venn diagram showing subgroup analysis of prescription pattern The Venn diagram shows the number (proportion) of the male and elderly participants prescribed with fixed-dose combination (FDC) and multiple anti-glaucoma drugs.

Figure 3 portrays the participants' prescription patterns. The most commonly prescribed drugs were beta-blockers (i.e., timolol; n = 78), followed by prostaglandin analogs (i.e., latanoprost, bimatoprost, and travoprost; n = 46), carbonic anhydrase inhibitors (i.e., dorzolamide, brinzolamide; n = 45), and alpha-1 sympathomimetics (i.e., brimonidine; n = 33). Drugs from all groups were only present in six prescriptions. Carboxymethyl cellulose (n = 6) and mannitol (n = 4) were other adjuvant medications. In each FDC analyzed, timolol was paired with either brimonidine, dorzolamide, or bimatoprost. Figure 4 displays the prescription trends for FDC and polytherapy among males and elderly patients. Forty-three prescriptions had FDC of drugs, and 61 had more than one drug. Only eight elderly males were prescribed multiple drugs, including FDC.

## Discussion

This prospective, observational study explored the drug utilization pattern of anti-glaucoma medications among those patients who visited the study site between January 2021 and February 2022 and met the study criteria. We assessed the range of prescribed anti-glaucoma medications and the proportion of patients receiving different medications. The percentage of patients who had polytherapy and FDC was also calculated. The most often administered medication, according to our observations, was timolol. Additionally, every FDC consistently included it.

Glaucoma was more commonly seen in women and older people in the study population than in their contemporaries. As acute angle closure glaucoma patients were surgically treated, most cases were ascertained to have primary open angle glaucoma. These findings were similar to the Bayesian meta-analysis by Rudnicka et al. [10] and the study by Allison et al. [11]. The average number of drugs per prescription was 1.75 (95% CI: 1.04-2.47), which concords with the findings of some previous studies [7,13-15]. Most participants (61%) had received combination therapy, which might have contributed to a more significant IOP reduction by different mechanisms. Our results agreed with three recent studies [6,8,16]. Most of the patient's prescriptions contained generic medications and clear instructions for the patients. Our study's findings agreed with those of other studies [6,8,9]. Most prescriptions included timolol. In each FDC analyzed, timolol was paired with either brimonidine, dorzolamide, or bimatoprost. The key considerations in choosing an anti-glaucoma medication were cost, patient compliance, and side effects of the eye drops.

In this study, we calculated the proportions of individuals who received monotherapy, polytherapy, or FDC (based on gender and age group). However, this study's results should be comprehended with a couple of limitations. First, there was a small sample size, presumably due to the nationwide outbreak of COVID-19. Second, the sample size was further trimmed by excluding comorbidities. Third, we could not perform cost-benefit and cost-effectiveness analyses since many patients did not report after procuring the prescribed medicines from outside sources. In contrast, some patients received the pharmaceuticals freely from the hospital supply.

## Conclusions

Timolol was identified as the most commonly prescribed medication for glaucoma and a key ingredient in the FDC. Doctors must rigorously abide by the national list of essential medicines and prescribe generic medications with clear instructions. Further studies encompassing pharmacoeconomic analysis are necessary.
